# The AUDANA algorithm for automated protein 3D structure determination from NMR NOE data

**DOI:** 10.1007/s10858-016-0036-y

**Published:** 2016-05-12

**Authors:** Woonghee Lee, Chad M. Petit, Gabriel Cornilescu, Jaime L. Stark, John L. Markley

**Affiliations:** National Magnetic Resonance Facility at Madison and Biochemistry Department, University of Wisconsin-Madison, Madison, WI 53706 USA; Department of Biochemistry and Molecular Genetics, University of Alabama at Birmingham, Birmingham, AL 35294 USA

**Keywords:** 3D structure determination, Automated structure calculation, NOE assignment, PACSY database, PONDEROSA, Sequence-structure correlation

## Abstract

We introduce AUDANA (Automated Database-Assisted NOE Assignment), an algorithm for determining three-dimensional structures of proteins from NMR data that automates the assignment of 3D-NOE spectra, generates distance constraints, and conducts iterative high temperature molecular dynamics and simulated annealing. The protein sequence, chemical shift assignments, and NOE spectra are the only required inputs. Distance constraints generated automatically from ambiguously assigned NOE peaks are validated during the structure calculation against information from an enlarged version of the freely available PACSY database that incorporates information on protein structures deposited in the Protein Data Bank (PDB). This approach yields robust sets of distance constraints and 3D structures. We evaluated the performance of AUDANA with input data for 14 proteins ranging in size from 6 to 25 kDa that had 27–98 % sequence identity to proteins in the database. In all cases, the automatically calculated 3D structures passed stringent validation tests. Structures were determined with and without database support. In 9/14 cases, database support improved the agreement with manually determined structures in the PDB and in 11/14 cases, database support lowered the r.m.s.d. of the family of 20 structural models.

**Electronic supplementary material:**

The online version of this article (doi:10.1007/s10858-016-0036-y) contains supplementary material, which is available to authorized users.

Three-dimensional structures of proteins provide important insights into their biological function. NMR spectroscopy is the sole approach for determining 3D structures of proteins in solution under near physiological conditions. In addition, NMR spectroscopy enables investigations of protein conformation and dynamics under different conditions. Whereas, structure determination from single-crystal X-ray diffraction has been largely automated, protein structure determination from NMR data still can require skilled manual intervention. This is particularly true for proteins that are large (>12 kDa), multimeric, or partially disordered. Most of the NMR-derived protein structures deposited in the Protein Data Bank (PDB) (Berman et al. 2009) represent monomeric proteins of fewer than 120 residues (Supplementary Fig. S1A). In addition, the number of NMR-derived structures is a small fraction of the total number of depositions (Supplementary Fig. S1B).

We have been developing an integrated approach to NMR-based protein structure determination that builds on *NMRFAM*-*SPARKY* (Lee et al. 2015), an updated and extended version of the highly popular *Sparky* program (Goddard and Kneller 2008). The *Integrative NMR* package (Lee et al. 2016) supports probabilistic methods for data interpretation (Bahrami et al. 2012; Lee et al. 2013) and automated structure determination from chemical shift assignments and NOE spectra (Lee et al. 2011). The structure determination package (*PONDEROSA*-*C/S*) (Lee et al. 2014) automates the identification of NOE cross peaks and the collection of torsion angle constraints. It also automates the data handling and format conversions required for use of the structure calculation modules of *CYANA* (Güntert 2004) and *Xplor*-*NIH* (Schwieters et al. 2003). The approach can flexibly incorporate data from other non-uniform sampling and reconstruction approaches (Dashti et al. 2015), such as *ist@HMS* (Hyberts et al. 2012) or *NESTA*-*NMR* (Sun et al. 2015).

Approaches have been introduced in recent years that take advantage of the growing number and variety of protein structures deposited in the Protein Data Bank (PDB) to assist in determining protein structures from NMR data. Shen and Bax (2007) introduced a method that employs SPARTA to refine fragment libraries used as input to Rosetta structure calculations (Shen et al. 2008). The CS-HM-Rosetta using 4D data has extended this approach to larger proteins (Thompson et al. 2012). The POMONA (protein alignments obtained by matching of NMR assignments) algorithm matches experimental chemical shifts to values predicted for the crystallographic database to generate templates for chemical shift-based Rosetta modeling. (Shen and Bax 2015). The CS23D (chemical shift to 3D structure) web server accepts chemical shifts and generates coordinates by means of homology modeling, chemical shift threading, or Rosetta-based shift-aided structure prediction (Wishart et al. 2008). Yet another bioinformatics approach combines sparse NMR data on a protein with distance restraints derived from evolutionary residue–residue couplings (Tang et al. 2015).

The AUDANA (Automated Database-Assisted NOE Assignment) algorithm introduced here (Fig. 1) improves the robustness of the *PONDEROSA*-*C/S* package by adding an alternative NOE assignment module that utilizes information from an enlarged version of PACSY database (Lee et al. 2012), which incorporates information on protein structures deposited in the Protein Data Bank (PDB). AUDANA extracts inter-proton contacts from structures of proteins with homologous sequences and compares them with possible distance constraints from the experimental 3D-NOE spectra; good matches serve to reinforce constraints (Fig. 2). AUDANA utilizes an endurance scoring system driven by probability and knowledge to carry out an improved analysis of the 3D-NOE data. In iterative structure calculations, added constraints that are consistent with improved structures are retained while that those that are not are abandoned.

**Fig. 1:**
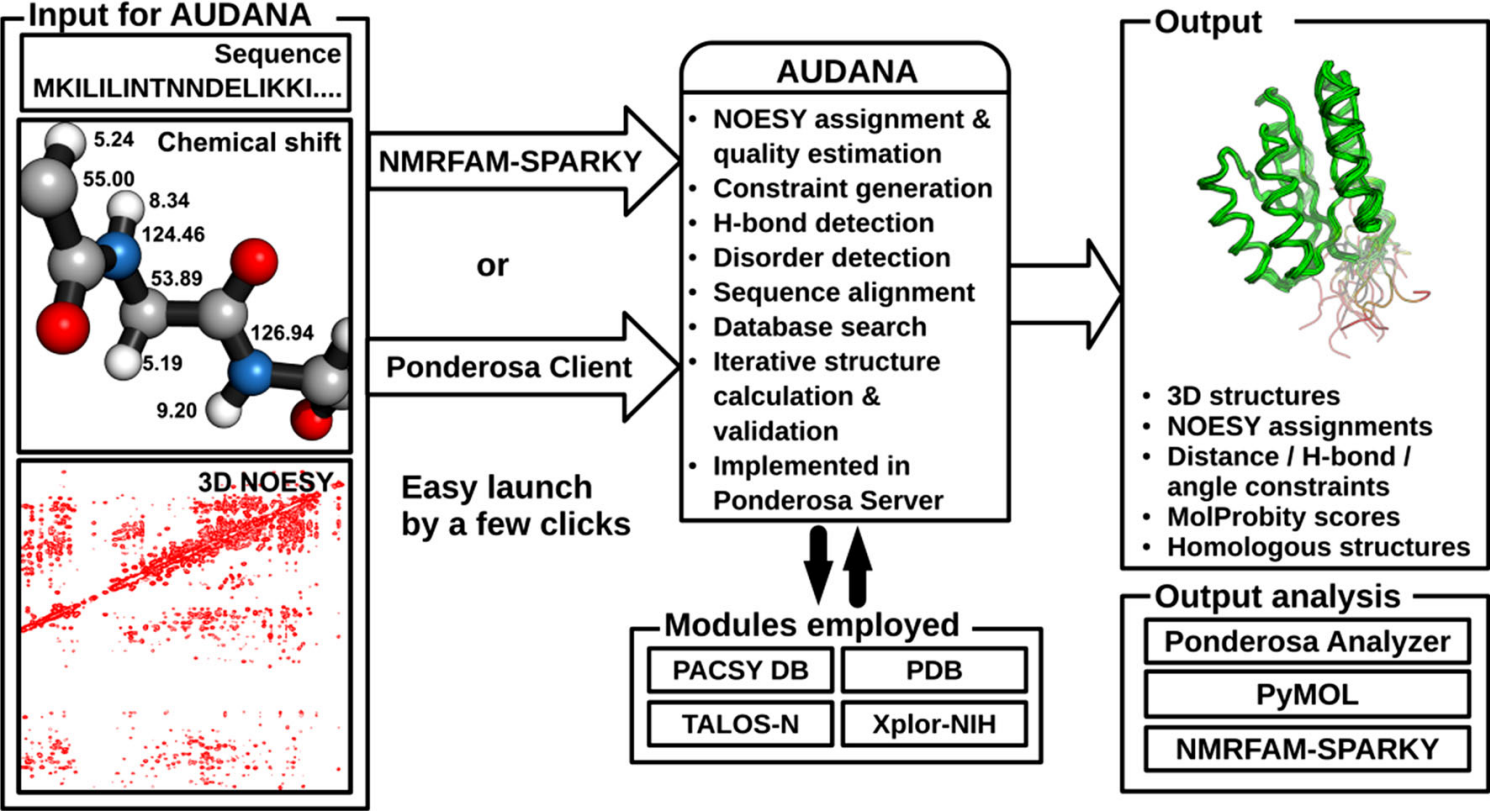
AUDANA is a new NOESY assignment algorithm for NMR based protein 3D structure determination that can be launched with a few clicks from either *NMRFAM*-*SPARKY* or *Ponderosa Client*. The AUDANA algorithm, which is carried out on the *Ponderosa Server*, employs the PACSY DB for bioinformatics, PDB files for 3D atom coordinates, *TALOS*-*N* for backbone angle constraints and order parameters from chemical shifts, and *Xplor*-*NIH* for simulated annealing. The results can be analyzed with the *PyMOL* and *NMRFAM*-*SPARKY* tools available as part of *Ponderosa Analyzer*

**Fig. 2:**
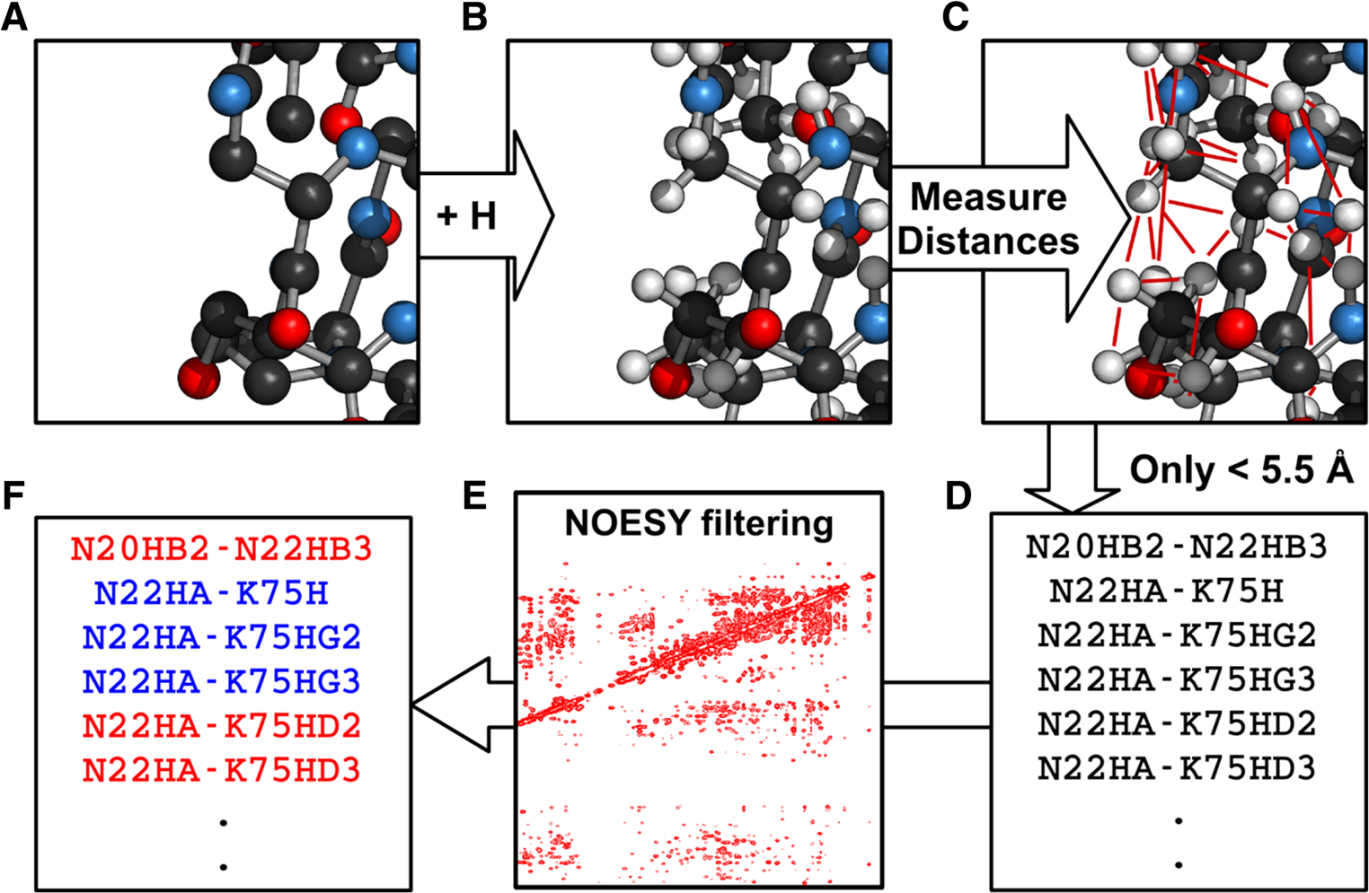
Illustration of how AUDANA extracts inter-proton contacts from structures of proteins with homologous sequences and compares them with possible distance constraints from the experimental 3D-NOE spectra. **a** A PDB model is chosen. **b** Hydrogens are added. **c** Interproton distances are calculated. **d** Distances less than 5.5 Å are tabulated. **e** Predicted NOE peaks are filtered against experimental data. **f** Those that match (blue) are retained; those that do not (red) are discarded

Initiation of a structure determination can be launched by two alternative methods: “AUDANA automation” or “PONDEROSA-X refinement”. The “AUDANA automation” optimizes user-supplied distance constraints, whereas the “PONDEROSA-X refinement” option runs AUDANA with automated NOESY assignments and torsion angle constraint optimization that automatically expands upper limits with elastic settings. By default, calculations are run on the NMRFAM-hosted *Ponderosa Server*. Users can run the software on their own hardware by installing the *Ponderosa Server*, the PACSY database, and the PACSY PDBSEQ_DB table expansion as described in Supplementary Table S1. AUDANA also can be launched directly from *NMRFAM*-*SPARKY* (Lee et al. 2015) by invoking “Calculation of 3D structure by *PONDEROSA*” (two-letter-code *c3*). The user then selects the NOESY spectra to be analyzed, and NOE cross peaks are identified automatically by the PONDEROSA algorithm. Alternatively, the user can submit NOE cross peaks chosen previously to the *Ponderosa Web Server* (http://ponderosa.nmrfam.wisc.edu/ponderosaweb.html). Structure calculations are carried out with the “PONDEROSA-X refinement” option, where “X” stands for *Xplor*-*NIH* annealing (Schwieters et al. 2003). Following the initial run, *Ponderosa Client* enables the user to add or modify constraints or change the calculation options.

AUDANA’s endurance scoring system consists of an endurance score, a supportive score, and a recycle bin. The endurance score for each distance constraint derived from NOESY data is determined initially by a statistical evaluation of the likelihood of its being correct. The endurance score is supplemented by the supportive score derived from finding similar structures in the database. The overall endurance score combines the supportive score with the endurance scores from NOESY data. The recycle bin is the place where violated distance constraints are temporarily stored. How they work together is described below.

AUDANA makes use of a queryable table “PDBSEQ_DB” (Supplementary Table S1) created by incorporating protein sequence data from the Protein Data Bank into the PACSY database. A total of 291,344 protein entries were included as of March 2016, and the resource is updated monthly. PDBSEQ_DB is available from the NMRFAM software download page (http://pine.nmrfam.wisc.edu/download_packages.html). By querying and aligning sequences from this table, AUDANA selects the three proteins with highest sequence homology to that of the target (Supplementary Fig. S2). Inter-proton distances determined from the structures of the homologous proteins are used to predict potential NOEs (Fig. 2); these predicted NOEs are filtered against the experimental NOESY data submitted by the user such that matches provide a supportive score for possible NOE assignments. However, if the sequence identity of the most similar protein is <20 %, no NOEs are predicted, and if it is >80 %, AUDANA uses only the structure of that single protein. The use of only one protein leads to a reduction in the supportive score and ensures that the structure of the target is not biased by that of the homolog because multiple sources of supportive score for the same constraint could be too high to be removed during the iterative structure calculation despite consistent violations.

AUDANA generates all possible combinations of distance constraints for each NOE cross peak by applying the “r^−6^—summed distance approximation”. Calculated endurance scores are used to evaluate the robustness of each assignment (Supplementary Fig. S3). Endurance scores for distance constraints from unambiguously assigned NOE cross peak are high, whereas those from ambiguously assigned peaks are low. Additional robustness is added by PACSY-derived supportive scores, which are based on the degree of local (tripeptide) match between the target and template sequence (Supplementary Fig. S4). Backbone angle constraints are calculated by *TALOS*-*N* (Shen and Bax 2013). Only “strong” and “generous” predictions from *TALOS*-*N* are used. 10° is used for all predicted deviations smaller than or equal to 10°; the value provided is used for predicted deviations between 11° and 35°, and 35° is used for all predicted deviations larger than 35°. The initial constraints for AUDANA are ± two times these angles for *strong* predictions and ± three times these angles for *generous* predictions. If an angle constraint is violated in 30 % (e.g. 6 out of 20) or more of the structures calculated in the “PONDEROSA-X refinement” option, the limits are expanded elastically in proportion to the average violation (*V*_*diff*_) and the number of structures in which the constraint was violated (*N*_*viol*_) according to the formula,

$${\text{Upper}}/{\text{lower}}\,{\text{limits}}\,(\theta_{N} )= {\text{Upper}}/{\text{lower}}\,{\text{limits}}\,\left( {\theta_{C} } \right) \pm 1.2 \times V_{diff} \times N_{viol} /20$$

where *θ*_C_ is the current limit and *θ*_N_ is the newly expanded limit. Structure calculation by AUDANA consists of 10,000 cycles of high-temperature (3500 °C) dynamics followed by low-temperature (25 °C) slow rigid-body simulated annealing carried out by the IVM module of *Xplor*-*NIH* (Schwieters et al. 2003). The set of distance constraints is updated after each iterative structure calculation (Supplementary Fig. S3D and Fig. 3a–c). In phase I, only constraints classified as “robust” with high endurance scores are used to calculate structures (Supplementary Fig. S3A); in phase II, “intermediate” level constraints are added; and in phase III, “uncertain” level constraints are added. In phases II and III, the lowest energy structure from the previous cycle is used to filter newly recruited constraints. Constraints in the recycle bin are checked after each cycle, and those that are not violated by the current model are recycled with the endurance score set to zero, such that they are readily removed if they are violated in subsequent cycles. After iterative runs of phases I to III, the best 20 models from phase III, are transferred to phase IV, where they are placed in water boxes and subjected to explicit water refinement with the final set of constraints (Fig. 3d).

**Fig. 3:**
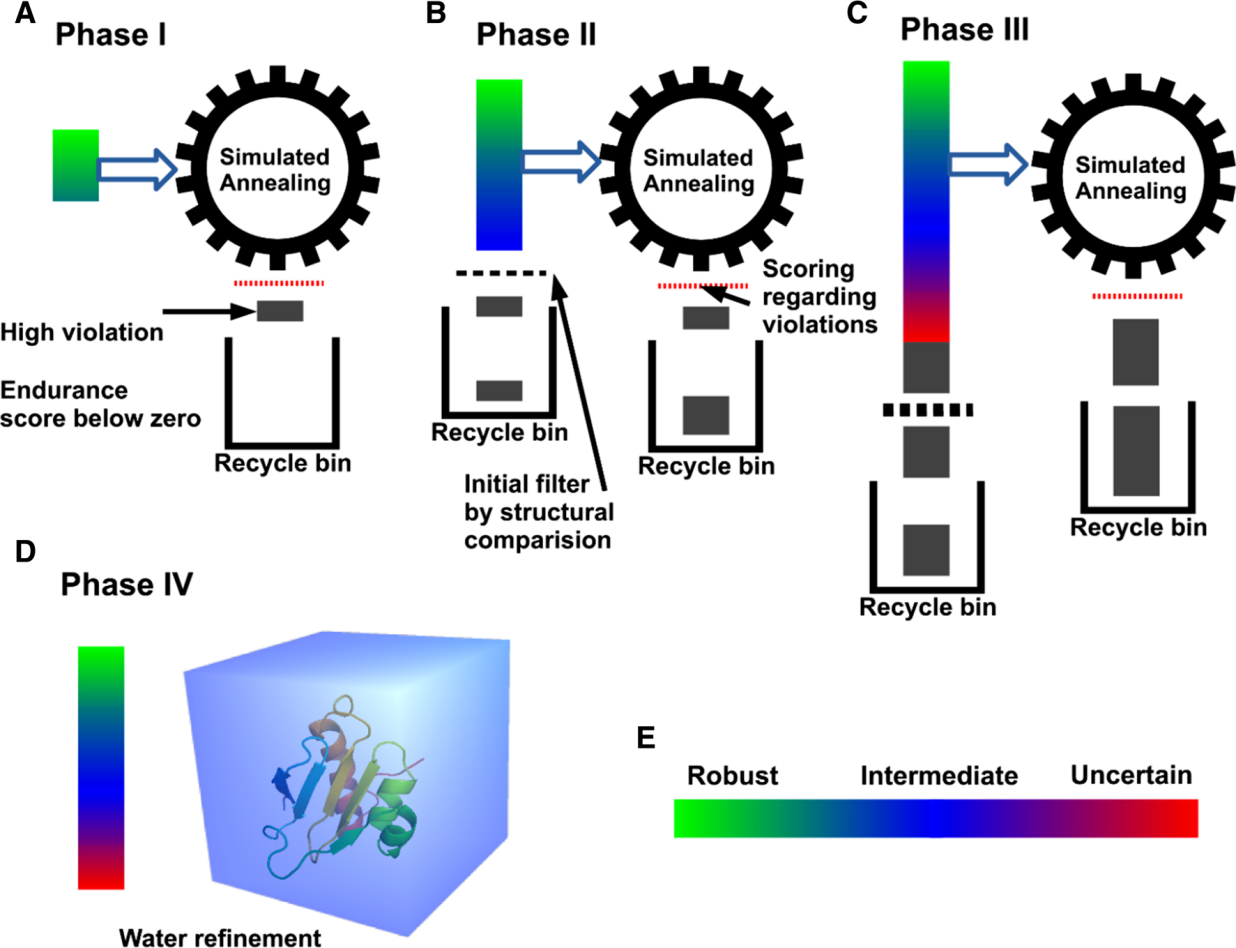
The four phases used by AUDANA in calculating NMR structure models and validating distance constraint sets. After completing each of phase I–III, constraints in the recycle bin are checked to determine if they are in agreement with the intermediate structure; if so, they are reincorporated into the constraint set but with zero endurance scores to make them susceptible to removal if they are violated in subsequent cycles. **a** In phase I, only distance constraints categorized as robust with high endurance scores are used for structure calculation. Highly violated constraints that score below zero after the score update go into the recycle bin. **b** In phase II, constraints in the intermediate category are included in the structure calculation. An initial filter based on the intermediate structure is applied to the constraints, and those that are strongly violated are removed. Constraints with endurance scores below zero after the update also go into the recycle bin. **c** In the phase III, constraints in the uncertain category are included; a more stringent filter against the intermediate structure is imposed. **d** Phase IV is explicit water refinement with the constraints from the last cycle. **e** Mapping of the robustness of constraints, color-coded by their endurance scores, onto the structure. **a, b, c** Dynamic hydrogen bond detection. During phase I, II and phase III, AUDANA detects potential hydrogen bonds from NOE cross peak patterns for secondary structures and generates idealized H-bond constraints for the calculation. After each calculation cycle, the H-bonds are reevaluated by measuring interatomic distances, and H-bond constraints that violate the structure are eliminated from use in the following cycle

During iterative structure calculation, AUDANA detects potential hydrogen bonds from NOE cross peak patterns for secondary structures and generates idealized H-bond constraints for the next cycle of calculation. After each calculation cycle, the H-bonds are reevaluated by measuring interatomic distances, and H-bond constraints that violate the structure are eliminated from use in the following cycle. *Ponderosa Server* automatically generates two *Xplor*-*NIH* constraint files from the H-bond constraints: the NOE constraint file, used to generate the NOE potential term (statically set to 30), and the HBDA constraint file, used for the HBDA potential term.

We tested AUDANA’s performance with data for 14 proteins (Supplementary Table S2). The *Ponderosa Client* program was used to import input data and run the calculations. To avoid biased cross validation, protein entries with identical sequences in the PACSY database were manually excluded from the sequence alignment process. Calculation options were set to “PONDEROSA-X refinement”, which runs AUDANA with torsion angle/rigid body dynamics and optimization by *Xplor*-*NIH*. We compared the lowest energy structure of each target to that of the first model deposited in the PDB (generally the representative structure with the lowest energy). All AUDANA calculated structures were very similar to those deposited in the PDB: the pairwise r.m.s.d. values for backbone atoms in ordered regions were less than 2 Å (mean r.m.s.d. of 1.41 ± 0.34 Å, Supplementary Table S2), and the superimposed structures were in close agreement (Supplementary Fig. S5). With these test proteins, AUDANA was instructed to select the best 20 out of 40 calculated structures at the phase III and IV. Targets considered difficult for automated NMR-based structure calculation, such as the symmetric homodimer NS1^RBD^ (Supplementary Fig. S5E) and the 25 kDa protein mThTPase (Supplementary Fig. S5 N) were solved successfully with backbone r.m.s.d. values to the deposited structure of 1.32 and 1.58 Å, respectively.

For comparison, we used AUDANA to determine the structures of the same 14 proteins without database assistance (this is accomplished by unchecking the “Use PACSY DB for better NOE assignment” option in the *Ponderosa Web Server*). The results (Supplementary Table S2, rightmost column) show that 5 of the 14 data sets, including that for the homodimer (NS1^RBD^) and the 25 kDa protein (mThTPase), failed to converge or had backbone r.m.s.d. values to the deposited structures greater than 2.0 Å. Two of these proteins have large disordered regions (HR6470A and HR5537A). Five proteins (with closest sequence identities 94, 62, 38, 33, and 33 %) yielded lower backbone r.m.s.d. values to the deposited structures without database support; however, three of these had aromatic NOESY and RDC data in addition to the usual ^13^C-NOESY and ^15^N-NOESY data. This suggests that additional experimental data can circumvent the need for database support.

Structural assessment was conducted by the *PSVS* package (Bhattacharya et al. 2007). Ramachandran plot analysis results from both *Procheck* (Laskowski et al. 1996) and *MolProbity* (Chen et al. 2015) were satisfactory (Supplementary Table S4). The option of calculating the best 20 out of 40 calculated models led to acceptable convergence of the ensembles (ensemble backbone r.m.s.d. values between 0.28 and 0.80 Å; except for 2.76 Å for mThTPase, Supplementary Table S2). By using the more rigorous “constraints only for the final step” option, which calculates the best 20 out of 100 models, the ensemble backbone r.m.s.d. for mThTPase was reduced to 1.81 Å (Supplementary Fig. S6).

*PONDEROSA*-*C/S* offers two options in “constraints only for the final step”: (1) the traditional method of explicit water refinement followed by simulated annealing, and (2) concurrent implicit water solvation with EEFx (Effective Energy Function for *Xplor*-*NIH*) potential during simulated annealing (Tian et al. 2014). We found that option 2 was frequently better at generating energetically favorable structures than option 1.

*Software availability* AUDANA is available from http://pine.nmrfam.wisc.edu/download_packages.html. Web server, instruction, manuals and video tutorials can be found at http://ponderosa.nmrfam.wisc.edu. AUDANA has been incorporated into the *PONDEROSA*-*C/S* web service at NMRFAM, which is freely available to academic users. AUDANA is incorporated into the *Integrative NMR* platform (Lee et al. 2016), which requires the installation of *NMRFAM*-*SPARKY*, *Ponderosa Analyzer*, *Ponderosa Client* and *PyMOL*. The website provides instructions, installation scripts and video tutorials for their installation. AUDANA is also incorporated into the *NMRFAM Virtual Machine* (Lee et al. 2016) which contains pre-installed versions of all relevant software. The virtual machine (VM) can be run under a number of different virtualization software programs (VirtualBox and VMware among others) that support the Open Virtualization Format (.ovf,.ova). These virtualization programs are available for a wide variety of different popular host computers and operating systems (Windows, Mac OSX, Linux). A VM emulates a complete computer system. For example, the base operating system of the *Integrative NMR* VM is Ubuntu Mate 15.04 (64 bit Linux) (https://ubuntu-mate.org); the virtualization software allows this Linux VM to run natively on any host computer.

## Electronic supplementary material

Below is the link to the electronic supplementary material.

Supplementary material 1 (PDF 2323 kb)
