# Supplementary material for: The AUDANA algorithm for automated protein 3D structure determination from NMR NOE data
Source: J Biomol NMR. 2016 May 12;65:51–7. doi: 10.1007/s10858-016-0036-y (PMC4921114; doi:10.1007/s10858-016-0036-y)
Supplement: Supplementary file 1 — Supplementary material 1 (PDF 2323 kb) [file 10858_2016_36_MOESM1_ESM.pdf]

**SUPPLEMENTARY INFORMATION**

# The AUDANA algorithm for automated protein 3D structure determination from NMR NOE data

Woonghee Lee,[1,*] Chad M. Petit,[2] Gabriel Cornilescu,[1] Jaime L. Stark,[1] John L. Markley[1,*]

[1]National Magnetics Resonance Facility at Madison and Biochemistry Department, University of Wisconsin-Madison, Madison, WI 53706, USA

[2]Department of Biochemistry and Molecular Genetics, University of Alabama at Birmingham, Birmingham, AL 35294, USA

* To whom correspondence should be addressed

E-mail: whlee@nmrfam.wisc.edu, Telephone: +1-608-263-1722

markley@nmrfam.wisc.edu, Telephone: +1-608-263-9349

**Supplementary Table S1.** Description of the PDBSEQ_DB table in the expanded version of the PACSY database (Lee et al. 2012). [1]

| Field | Type | Null | Key | Default | Extra |
|---|---|---|---|---|---|
| ID | INT(11) | YES | - | NULL | - |
| PDB_ID | CHAR(5) | NO | MUL | NULL | - |
| CHAIN_ID | CHAR(1) | NO | MUL | NULL | - |
| SEQ_COUNT | INT(11) | NO | MUL | NULL | - |
| SEQUENCE | TEXT | YES | - | NULL | - |

[1] 291,344 protein entries were included as of March 2016. The “genpdbseq_db.py” script available from the NMRFAM software download page (http://pine.nmrfam.wisc.edu/download_packages.html) automatically generates this table. As it can be seen in the Key column, PDB_ID, CHAIN_ID and SEQ_COUNT fields are indexed for performance. Default data for all the fields are set to NULL. Auto increment, time stamp, or other virtual generated data are not set as it can be seen in Extra column.

**Supplementary Table S2.** AUDANA results for the 14 test proteins.

| Targets | PDB ID | BMRB ID | Size | Similar protein (% identity) | Observed regions | Backbone r.m.s.d. [4] | All heavy atom r.m.s.d. [4] | Backbone r.m.s.d. (no database support)[4] |
|---|---|---|---|---|---|---|---|---|
| Brazzein [1,7] | 2LY5 | 16215 | 53 | 2KYQ (94) | 4-51 | 1.05 (0.68) | 1.35 (1.29) | 1.00 (0.72) |
| StT322 [3,5] | 2LOJ | 18214 | 63 | 4YNX (62)<br>1V1H (35)<br>2QBP (31) | 39-62 | 1.61 (0.38) | 1.96 (0.83) | 1.42 (0.42) |
| HR6470A [2,5] | 2L9R | 17484 | 69 | 1FTT (61)<br>1VND (61)<br>3A01 (49) | 13-58 | 1.52 (0.54) | 1.89 (1.12) | 2.71 (1.73) |
| HR8254A [3,5] | 2M2E | 18909 | 73 | 2CQR (37)<br>2YUM (39)<br>2CJJ (31) | 6-60 | 1.41 (0.49) | 1.67 (1.03) | 1.60 (0.35) |
| NS1RBD [2,7] | 2N74 | 25793 | 73 | 2Z0A (85) | 3-71 | 1.32 (0.38) | 1.66 (1.19) | 9.07 (4.71) |
| Ubiquitin [1,7] | 1D3Z | 6457 | 76 | 5AF6 (98) | 2-71 | 1.16 (0.41) | 1.38 (0.94) | 2.09 (0.92) |
| OR135 [2,5] | 2LN3 | 18145 | 83 | 2LTA (38)<br>2LVB (34)<br>2LND (34) | 5-73 | 1.13 (0.30) | 1.47 (0.84) | 1.03 (0.19) |
| HR2876C [3,5] | 2M5O | 19068 | 97 | 1VEH (85) | 17-91 | 1.19 (0.35) | 1.43 (0.73) | 1.71 (0.53) |
| HR6430A [2,5] | 2LA6 | 17508 | 99 | 2LCW (83) | 15-53,64-97 | 0.71 (0.28) | 0.84 (0.81) | 0.89 (0.63) |
| HR2876B [2,5] | 2LTM | 18489 | 107 | 2LTL (33)<br>2K1H (37)<br>2VU5 (27) | 13-28,38-100 | 1.90 (0.80) | 2.03 (1.19) | 1.22 (0.90) |
| YR313A [2,5] | 2LTL | 18487 | 119 | 2LTM (33)<br>3L7X (33)<br>4IWB (28) | 19-22,29-34,48-88,94-111 | 1.59 (0.72) | 2.10 (1.01) | 1.52 (0.61) |
| OR36 [2,5] | 2LCI | 17613 | 134 | 2LR0 (98) | 3-128 | 1.75 (0.40) | 2.05 (1.01) | 2.00 (0.51) |
| HR5537A [1,6] | 2KK1 | 16349 | 135 | 1ZZP (45)<br>4GE3 (28)<br>4QYT (28) | 39-106,117-134 | 1.86 (0.72) | 2.25 (1.28) | 2.80 (3.63) |
| mThTPase [3,7] | 2JMU | 15063 | 224 | 5A65 (96) | 6-66,79-96,106-182,193-213 | 1.58 (2.76) | 2.18 (3.02) | N/A (N/A)[8] |

[1] Inputs used: sequence, chemical shifts, $^{13}$C-NOESY and $^{15}$N-NOESY.
[2] Inputs used: sequence, chemical shifts, $^{13}$C-NOESY, $^{15}$N-NOESY, aromatic NOESY, and RDC data.
[3] Inputs used: sequence, chemical shifts, $^{13}$C-NOESY, $^{15}$N-NOESY, and aromatic NOESY.
[4] The numbers outside parentheses are the r.m.s.d. between the PDB deposited model and the best AUDANA model; the numbers in parentheses are the pairwise r.m.s.d. for the 20 structural models.
[5] CASD-NMR 2013 targets (Rosato et al. 2015). Inputs were acquired from CASD-NMR web page (https://www.wenmr.eu/wenmr/casd-nmr-data-sets).
[6] CASD-NMR 2010 targets (Rosato et al. 2009) Inputs were acquired from CASD-NMR web page (https://www.wenmr.eu/wenmr/casd-nmr-data-sets).
[7] NMRFAM internal or collaborative projects (Cornilescu et al. 2013, Jureka et al. 2015, Song et al. 2008).
[8] The *Ponderosa Server* does not proceed to the water refinement if the backbone pairwise r.m.s.d. of the 20 structural models failed to converge under 10 Å as in this case.

**Supplementary Table S3.** Numbers of distance and angle constraints remaining at the end of phases I-III (Fig. S6 ABC) and used for the final structure calculations. Database-supported constraints were used only in determining endurance scores.

| Targets | Intra $i = j$ | Sequential $\|i - j\| < 2$ | Medium $2 \leq \|i - j\| < 5$ | Long $5 \leq \|i - j\|$ | Total from NOEs | Angle constraints [1] $\varphi$ / $\psi$ | Database supported |
|---|---|---|---|---|---|---|---|
| Brazzein | 429 | 204 | 226 | 217 | 1076 | 30 / 26 | 289 |
| StT322 | 335 | 146 | 115 | 230 | 826 | 29 / 31 | 157 |
| HR6470A | 462 | 212 | 370 | 188 | 1232 | 40 / 42 | 259 |
| HR8254A | 766 | 335 | 496 | 189 | 1786 | 57 / 62 | 253 |
| $NS1^{RBD}$ | 200 | 151 | 366 | 104 | 821 | 62 / 64 | 220 |
| Ubiquitin | 624 | 276 | 284 | 437 | 1621 | 49 / 55 | 464 |
| OR135 | 1052 | 468 | 452 | 406 | 2378 | 67 / 63 | 7 |
| HR2876C | 869 | 467 | 624 | 518 | 2478 | 47 / 52 | 738 |
| HR6430A | 553 | 340 | 315 | 692 | 1900 | 57 / 53 | 747 |
| HR2876B | 1143 | 453 | 411 | 420 | 2427 | 65 / 75 | 32 |
| YR313A | 1221 | 496 | 400 | 364 | 2481 | 70 / 69 | 46 |
| OR36 | 1301 | 619 | 666 | 778 | 3364 | 103 / 94 | 1041 |
| HR5537A | 497 | 382 | 692 | 87 | 1658 | 71 / 80 | 102 |
| mThTPase | 609 | 437 | 400 | 558 | 2004 | 127 / 139 | 571 |

[1] Angle constraints are predicted by *TALOS-N* (Shen and Bax 2013) and optimized by AUDANA.

**Supplementary Table S4.** Structural quality assessment report from the PSVS package (Bhattacharya et al. 2007).

| Targets | Procheck Validation | | | | | MolProbity Validation | | | | Num. of close contact |
|---|---|---|---|---|---|---|---|---|---|---|
| | A | B | C | D | Procheck [1,3] φ-ϕ | E | F | G | Clashscore [2,4] | - |
| Brazzein | 77.8 | 18.3 | 1.7 | 2.2 | -0.48 (-1.57) | 88.0 | 10.0 | 2.0 | 60.31 (-8.82) | 9 |
| StT322 | 84.8 | 14.1 | 1.1 | 0.0 | -0.71 (-2.48) | 91.7 | 4.2 | 4.2 | 42.45 (-5.76) | 4 |
| HR6470A | 93.3 | 6.0 | 0.4 | 0.2 | 0.29 (1.46) | 96.1 | 3.7 | 0.2 | 47.52 (-6.63) | 15 |
| HR8254A | 97.5 | 2.5 | 0.0 | 0.0 | 0.59 (2.64) | 98.4 | 1.6 | 0.0 | 40.44 (-5.41) | 14 |
| $NS1^{RBD}$ | 97.5 | 2.5 | 0.0 | 0.0 | 0.64 (2.83) | 99.9 | 0.1 | 0.0 | 35.72 (-4.60) | 5 |
| Ubiquitin | 93.4 | 4.5 | 2.0 | 0.1 | -0.30 (-0.87) | 96.6 | 1.6 | 1.9 | 59.84 (-8.74) | 40 |
| OR135 | 94.2 | 5.8 | 0.0 | 0.0 | 0.06 (0.55) | 98.3 | 1.7 | 0.0 | 66.94 (-9.96) | 18 |
| HR2876C | 85.8 | 12.3 | 1.9 | 0.0 | -0.33 (-0.98) | 92.4 | 6.7 | 0.9 | 79.82 (-12.17) | 39 |
| HR6430A | 93.6 | 5.0 | 0.0 | 1.4 | -0.16 (-0.31) | 96.8 | 2.2 | 1.0 | 65.45 (-9.71) | 7 |
| HR2876B | 94.3 | 4.6 | 0.7 | 0.4 | -0.14 (-0.24) | 97.3 | 2.4 | 0.3 | 46.13 (-6.39) | 9 |
| YR313A | 89.2 | 9.6 | 0.9 | 0.2 | -0.25 (-0.67) | 94.2 | 5.1 | 0.7 | 48.87 (-6.86) | 25 |
| OR36 | 94.7 | 5.3 | 0.0 | 0.0 | 0.07 (0.59) | 98.5 | 1.5 | 0.0 | 105.13 (-16.51) | 66 |
| HR5537A | 97.8 | 2.1 | 0.1 | 0.1 | 0.48 (2.20) | 98.1 | 1.9 | 0.0 | 33.79 (-4.27) | 19 |
| mThTPase | 90.7 | 8.3 | 0.3 | 0.6 | -0.16 (-0.31) | 96.2 | 2.7 | 1.1 | 40.89 (-5.49) | 35 |

**A** Most favored regions (%)
**B** Additionally allowed regions (%)
**C** Generously allowed regions (%)
**D** Disallowed regions (%)
**E** Most favored regions (%)
**F** Allowed regions (%)
**G** Disallowed regions (%)

[1] (Laskowski et al. 1996)
[2] (Chen et al. 2015)
[3,4] Raw score (Z-score)

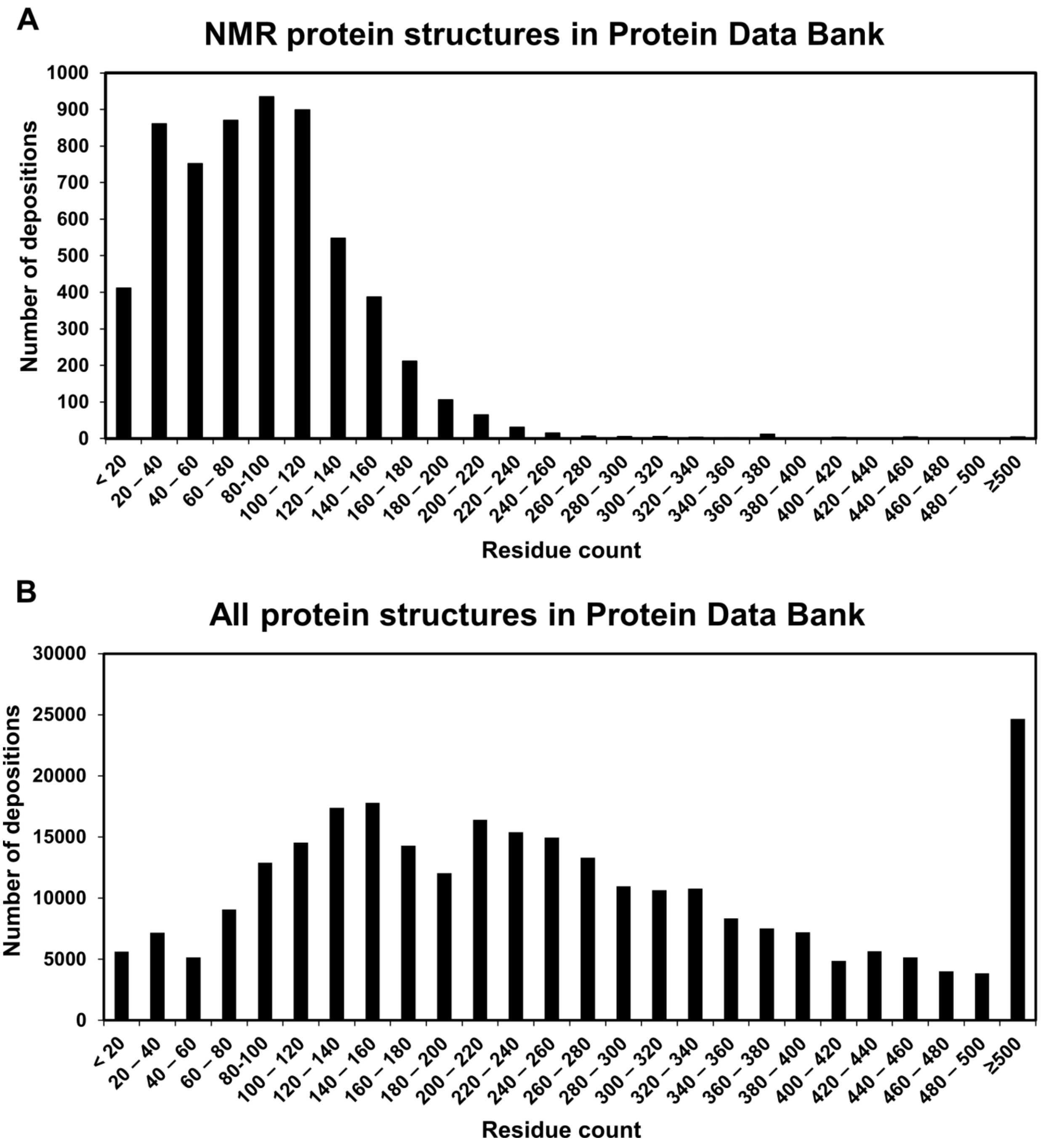


**Supplementary Fig. S1.** Bar graph illustrating 3D structures deposited in Protein Data Bank (Berman et al. 2007) as of October 2015. **A.** NMR structures deposited in PDB as queried from the PACSY DB (Lee et al. 2012). **B.** All structures in PDB regardless of determination method.

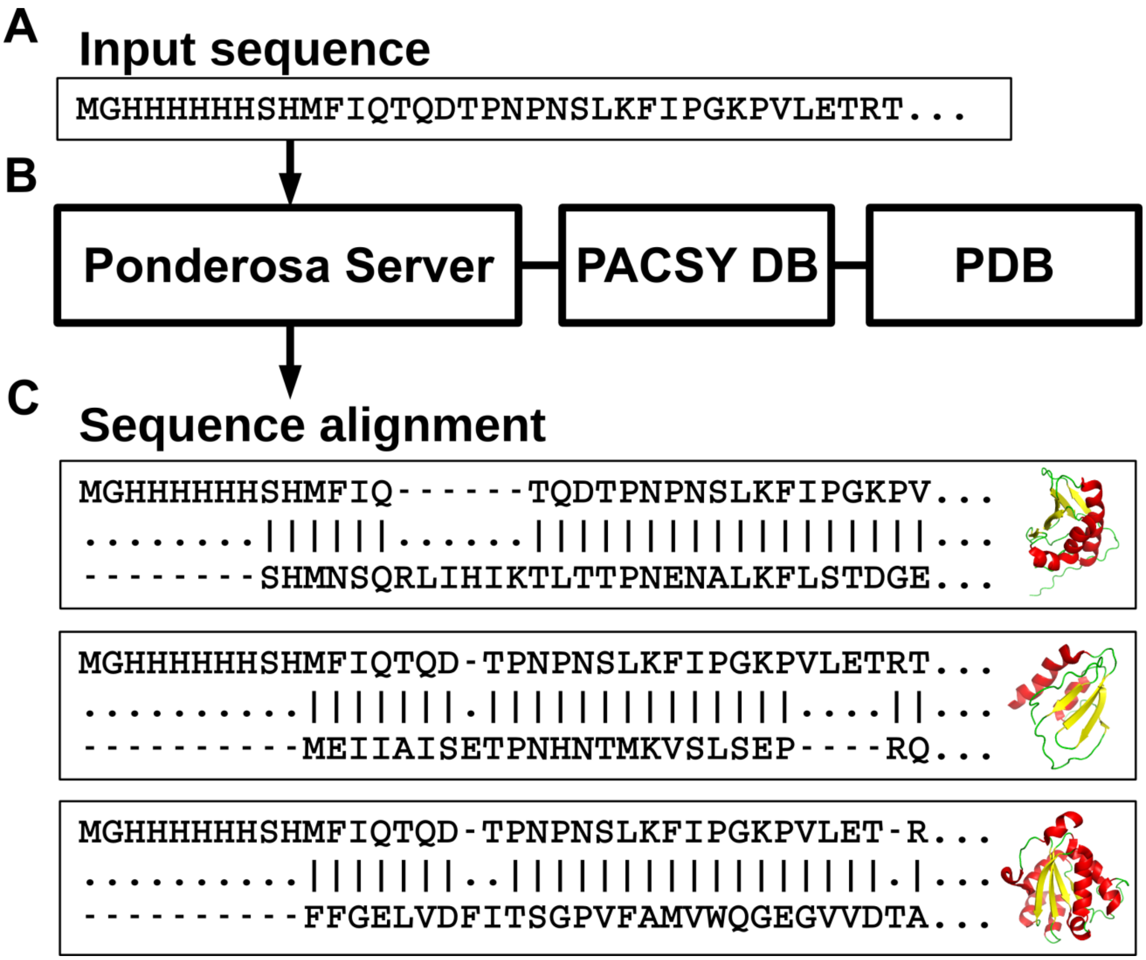


**Supplementary Fig. S2.** AUDANA selects the three most similar proteins by aligning the input sequence from the user to sequences in the PACSY database. **A.** The user sequence is entered. **B.** The PDBSEQ_DB table in PACSY is queried to restrict the pool of sequences from PDB to the size of the input sequence to achieve better performance. **C.** The *Ponderosa Server* aligns sequences by the Smith-Watermann algorithm (Smith and Waterman 1981) to determine the three best matches.

**A**

$$P_k = \frac{E_{k1} + E_{k2} + E_{k3} + E_{k4}}{\sum (E_{k1} + E_{k2} + E_{k3} + E_{k4})}$$

$P_k$ : Probability of $k$th inter-proton contact from experimental NOE data.

$E_{kn}$ : Evidence for the $n$th category for the $k$th probable inter-proton contact from NOE cross peaks.

**B**

$$S_k = C_R \times P_k$$

$S_k$ : Endurance score for $k$th probable inter-proton contact.

$C_R$ : Constant set to 20.

**C**

$$S_{TOT} = S_N + S_C + S_A + S_P$$

$S_{TOT}$ : Overall endurance score.

$S_{N,C,A}$ : Endurance scores calculated from N-NOESY, C-NOESY, or aromatic NOESY.

$S_P$ : Supportive scores from the overall identity between the tripeptide centered on the residue of interest and the tripeptide from the homologous protein.

**D**

$$S_{NEW} = S_{OLD} - S_{VIOL}$$

$S_{NEW}$ : Updated endurance score after a cycle of structure calculation.

$S_{OLD}$ : The endurance score in the previous cycle.

$S_{VIOL}$ : Deducting arisen from violation in the structure calculation.

**Supplementary Fig. S3.** Equations used in determining the endurance score. **A.** Probabilities are calculated for each potential inter-proton constraint corresponding to an NOE peak from: 1) the network of potential interactions involving a pair of residues supported by NOE data ($E_{k1}$); 2) probability for hydrogen atoms from the particular residue pair being within 5.5 Å ($E_{k2}$); 3) order parameters as calculated from chemical shifts (Berjanskii and Wishart 2005) ($E_{k3}$); and 4) agreement between the position of the NOE peak and the assigned chemical shifts ($E_{k4}$). **B.** The endurance score for the candidate inter-proton constraint from a NOE peak is defined as the product of a constant ($C_R$) and the probability for the candidate ($P_k$). The constant 20 was initially picked randomly and other values were optimized depending on the number. Constraints with low probability are vulnerable to being removed in score updates (Supplementary Fig. S4D). **C.** The total endurance score used in a structure calculation is the sum of the scores from different types of experimental data ($S_N$, $S_C$ and $S_A$) and the supportive score ($S_P$, explained in Supplementary Fig. S5). **D.** After each structure calculation cycle, violations are examined, and ($S_{VIOL}$) is deducted from the endurance score of the completed cycle ($S_{OLD}$) to yield an updated score ($S_{NEW}$). The deduction score is dynamically scaled product of the number of violated structures, the violated distances and the phase (Supplementary Fig. S6). If the new score is less than zero, the constraint goes into the recycle bin. If the violated constraint survives, the upper distance is extended elastically. The process is illustrated in Supplementary Fig. S6.

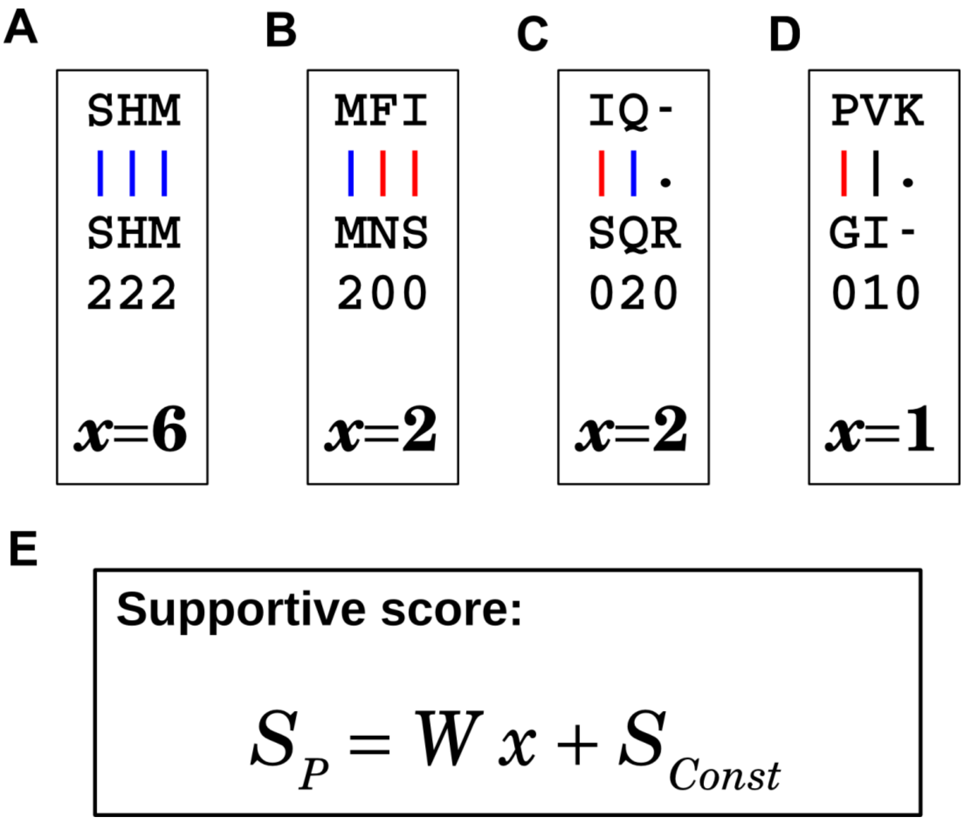


**Supplementary Fig. S4.** Equations used in determining the supportive score. The supportive score to be added to the endurance score from the experimental data is calculated from the knowledge-based inter-proton contact list (Fig. 2). The score for a residue is determined from the similarity between tri-peptides (with the residue in question as the central residue) from the user sequence and the aligned sequence from PDB. The score for each exactly matched residue is 2; the score for a similar residue is 1; and dissimilar matches or gaps are scored as 0. The scores from the three residues in the tripeptide are summed yield the multiplication factor (x). **A**. Example in which all three aligned residues match; the multiplication factor (x) is 6. **B** and **C.** Examples in which only one residue matches; x=2 in each case. **D.** Example in which two residues do not match, but the third is similar; x=1. **E.** The supportive score for the constraint is calculated as a linear function of the multiplication component (*x*) with slope W (empirical weighting factor); the x-axis intercept is the minimum supportive score ($S_{const}$) for the aligned sequence. Currently, $W$ is 2.5 and $S_{const}$ is 15. The individual parameters may be improved as they are empirically driven. However, this setup has worked fairly well so far.

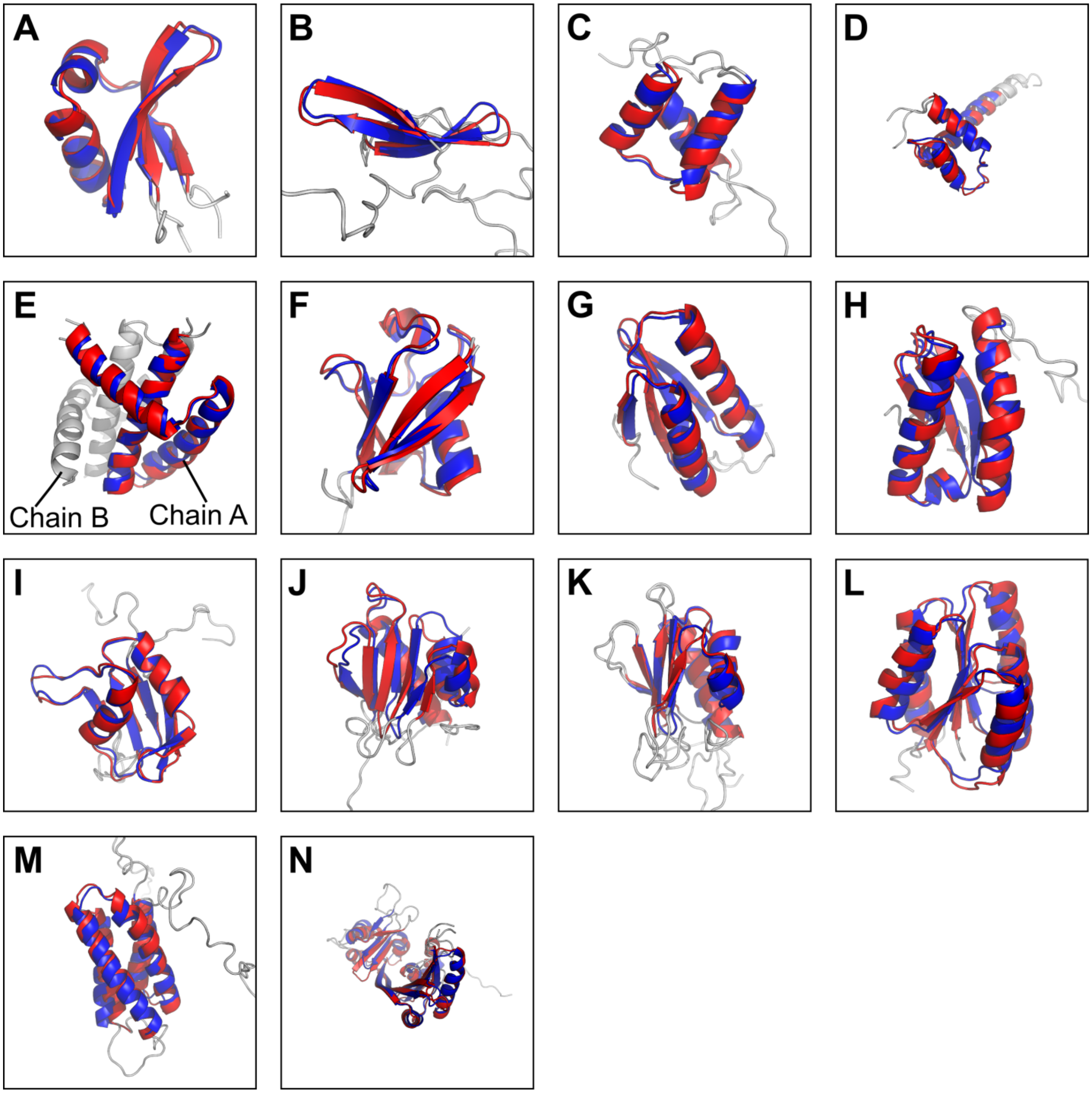


**Supplementary Fig. S5.** Superimpositions of AUDANA structure (blue) and PDB deposited structure (red) for the 14 protein targets. All the targets calculated by AUDANA were close to those deposited in the PDB (< 2 Å r.m.s.d. for backbone atoms, Supplementary Table S2). **A.** Brazzein. **B.** StT322. **C.** HR6470A. **D.** HR8254A. **E.** NS1$^{RBD}$ (monomeric domain of the symmetric homodimer). **F.** Ubiquitin. **G.** OR135. **H.** HR2876C. **I.** HR6430A. **J.** HR2876B. **K.** YR313A. **L.** OR36. **M.** HR5537A. **N.** mThTPase (25-kDa).

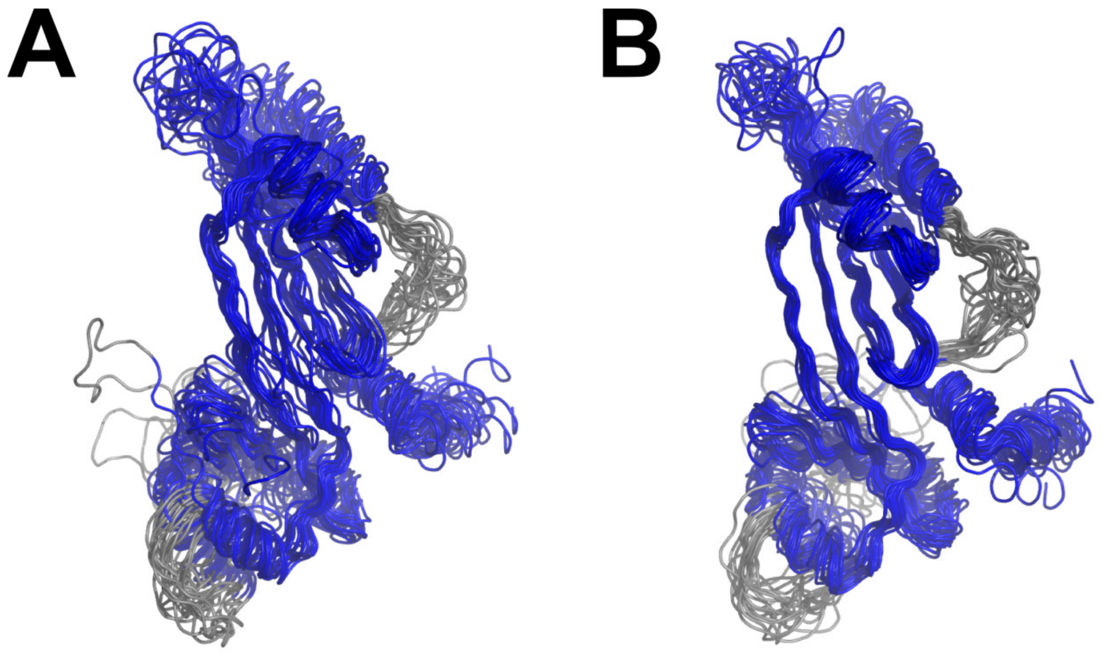


**Supplementary Fig. S6.** Comparison of the structural ensembles obtained for mThTPase (25 kDa protein) by using the “PONDEROSA-X refinement” and “constraint only for the final step” options. A. Selected the best 20 out of 40 calculated structures from “PONDEROSA-X refinement option”. Pairwise backbone atom r.m.s.d. for the structural models is 2.76 Å. B. Selected the best 20 out of 100 calculated structures from *traditional* “constraint only for the final step”. Pairwise backbone atom r.m.s.d. for the structural models is reduced to 1.81 Å.

## REFERENCES


Berjanskii MV, Wishart DS (2005) A simple method to predict protein flexibility using secondary chemical shifts. J Am Chem Soc 127:14970-14971

Berman H, Henrick K, Nakamura H, Markley JL (2007) The worldwide Protein Data Bank (wwPDB): ensuring a single, uniform archive of PDB data. Nucleic Acids Res 35:D301-303

Bhattacharya A, Tejero R, Montelione GT (2007) Evaluating protein structures determined by structural genomics consortia. Proteins 66:778-795

Chen VB, Wedell JR, Wenger RK, Ulrich EL, Markley JL (2015) MolProbity for the masses-of data. J Biomol NMR 63:77-83

Cornilescu CC, Cornilescu G, Rao H, Porter SF, Tonelli M, Derider ML, Markley JL, Assadi-Porter FM (2013) Temperature-dependent conformational change affecting Tyr11 and sweetness loops of brazzein. Proteins 81:919-925

Jureka AS, Kleinpeter AB, Cornilescu G, Cornilescu CC, Petit CM (2015) Structural Basis for a Novel Interaction between the NS1 Protein Derived from the 1918 Influenza Virus and RIG-I. Structure 23:2001-2010

Laskowski RA, Rullmann JAC, MacArthur MW, Kaptein R, Thornton JM (1996) AQUA and PROCHECK-NMR: Programs for Checking the Quality of Protein Structures Solved by NMR. J Biomol NMR 8:477-486

Lee W, Yu W, Kim S, Chang I, Lee W, Markley JL (2012) PACSY, a relational database management system for protein structure and chemical shift analysis. J Biomol NMR 54:169-179

Rosato A, Bagaria A, Baker D, Bardiaux B, Cavalli A, Doreleijers JF, Giachetti A, Guerry P, Guntert P, Herrmann T, Huang YJ, Jonker HR, Mao B, Malliavin TE, Montelione GT, Nilges M, Raman S, van der Schot G, Vranken WF, Vuister GW, Bonvin AM (2009) CASD-NMR: critical assessment of automated structure determination by NMR. Nat Methods 6:625-626

Rosato A, Vranken W, Fogh RH, Ragan TJ, Tejero R, Pederson K, Lee HW, Prestegard JH, Yee A, Wu B, Lemak A, Houliston S, Arrowsmith CH, Kennedy M, Acton TB, Xiao R, Liu G, Montelione GT, Vuister GW (2015) The second round of Critical Assessment of Automated Structure Determination of Proteins by NMR: CASD-NMR-2013. J Biomol NMR

Shen Y, Bax A (2013) Protein backbone and sidechain torsion angles predicted from NMR chemical shifts using artificial neural networks. J Biomol NMR 56:227-241

Smith TF, Waterman MS (1981) Identification of common molecular subsequences. J Mol Biol 147:195-197

Song J, Bettendorff L, Tonelli M, Markley JL (2008) Structural basis for the catalytic mechanism of mammalian 25-kDa thiamine triphosphatase. J Biol Chem 283:10939-10948
